# The effects of lockdown of work and activities for adults with multiple, complex needs including sensory impairments during the pandemic in 2020

**DOI:** 10.1177/17446295241232030

**Published:** 2024-02-02

**Authors:** Trine Lise Bakken, Bodil Ellingsen

**Affiliations:** National Advisory Unit for mental health in Intellectual Disability, 55272 Oslo University Hospital, Norway; The Signo Vivo Centre, Norway

**Keywords:** sensory loss, intellectual disabilities, complex and multiple needs, COVID-19 pandemic

## Abstract

Sheltered work and leisure activities were locked down in at the Signo centre in March 2020 because of the COVID-19 pandemic. The Signo centre is a Norwegian national centre for adults with multiple, complex needs, including severe sensory loss/impairments. Tension and uncertainty rapidly spread among relatives and workers. To explore the impacts of the pandemic on residents, 24 adults living in Signo Vivo answered a semi-structured interview together with their primary worker. Additionally, reports on staff injuries and PRN medication between April and Aug of 2020 were compared to the period before the lockdown. The reports from the interviews included fewer stressful events for the participants, more rest and sleep, more time spent in their own apartments, and more time with smaller groups of workers. The reports on staff injuries and PRN medication showed decreased occurrence.

## Introduction

Norway closed down large sections of the community during the COVID-19 pandemic in March 2020. People with intellectual disabilities (ID) constitute a largely heterogeneous group, ranging from people with borderline IQ, to people with more severe ID, and additional autism spectrum disorder, mental disorders, vision and/or hearing impairments (sensory loss), and physical disorders. Dysfunctional sensory processing and/or sensory loss, as well as mental disorders occur frequently in those with both autism and ID (Bakken and Sageng, 2016; Fellinger et al., 2009). Autistic people with mental ill-health, and a variety of conditions mentioned above additional to ID constitute a sub-group of people with ID and multiple, complex needs (Cook and Hole, 2021; Rosengard et al., 2007).

Knowledge about mental health in people with ID and additional sensory loss is an understudied area (Kildahl et al., 2019; Verdier et al., 2020). Sensory disabilities may in themselves cause mental ill-health (Augestad, 2017; Bodsworth et al., 2011; Dammeyer and Chapman, 2017; Basillers, 1973). A Danish population study found that people with sensory loss have increased risk of developing both mental ill-health and physical disorders. This study also found that there is an elevated risk of having ID in people with sensory loss compared to the general population (Dammeyer and Chapman, 2017). A Norwegian case study including two young adults with congenital blindness, co-occurring autism and ID emphasises that assessment and treatment of mental disorders in this group are especially complicated, because most of the material made for stress regulation is visual (Kildahl et al., 2019).

People with ID in Norway found that sheltered work facilities, day centres and leisure activities were closed, or access was limited during the COVID-19 pandemic. As in other countries, infection control measures resulted in strict regulations regarding social mobility, including activities for people with ID who depend on community services (Bigby, 2020). A newly published review found increased stress levels, anxiety and isolation in people with ID during the pandemic (Lunsky et al., 2022). Most user experiences during the pandemic for the most part support the Lunsky review. An online study from the USA which investigated 181 adults with ID in a convenience sample found that the pandemic mostly impacted the participants negatively regarding job loss, social isolation, and elevated stress levels (Fisher et al., 2021). A qualitative study from South Korea investigated the reactions of 15 adults with mild or moderate ID who lost access to their community services: even though this situation had a negative impact on daily routines, family relationships and social life of the participants of the study, they also developed new ways of adapting in difficult situations nevertheless (Kim et al., 2021). A UK clinical study investigated the impact of the pandemic on 46 adults with Down Syndrome (DS). One out of five participants lived at home. In line with the studies mentioned above, the study showed a negative impact from the pandemic on well-being and social life, and depressive symptoms (Villani et al., 2020).

An Italian study including 138 older adults with ID and autism in two group homes found, contrary to the studies mentioned above, no signs of increased stress levels or other behaviour changes (Raggi et al., 2013; Santambrogio et al., 2021). A Norwegian study found that users with multiple, complex needs in ten group homes appeared to experience less stress during the pandemic, contrary to the expectations of their workers (Hørsrud and Bakken, 2022). The findings in this study indicated more well-being among users. It was noted that receiving services at home, largely flexible schedules, staying in bed longer in the morning, and avoiding stressful situations such as travelling in minibuses, were appreciated by the users.

Services are provided for Norwegian adults with multiple, complex needs including sensory loss/impairments at a national centre, Signo Vivo, situated about 150 kilometres south-west of the capital, Oslo. This centre provides group homes, sheltered workplaces, and leisure activities for people with severe or complete sensory loss of vision or hearing, or both, and additional cognitive impairments. The Signo Vivo has three group homes for about 75 residents altogether. The centre provides sheltered work, as well as leisure activities. The staff members are special-need pedagogues, mental health nurses etc. The staff members master Braille and sign language / signs for speech. They also master facilitation of services for people with severe vision impairments.

The present study includes users with ID and multiple, complex needs, living in one group home at the national centre, Signo Vivo. The initiative for the study was taken by two of the professional staff counsellors at the centre, as they learned a couple of months into the pandemic that there were fewer reports of ad-hoc medication used for stress and anxiety regulation, as well as fewer incidents of users behaving aggressively to staff members.

To our knowledge, no studies on pandemic impacts of intellectual disability and/or autism and sensory disabilities have been done. Contrary to general findings of these disabilities separately that the pandemic led to increased stress, two staff from The Signo Vivo, an institutional setting in Norway supporting adults with both sensory and ID / autism, observed fewer reports of PRN medication use and behaviour incidents in the early part of the pandemic. The Signo Vivo organization was interested in exploring more systematically what the impacts of closures were on residents, to see if in fact, people were struggling less than pre-pandemic.

The aim of the study was to investigate how the close-down of sheltered workplaces and leisure activities impacted residents at the Signo Vivo in one group home during the first four months of the lockdown from March 2020. Two research questions were asked:

1. How did the residents experience the close-down of sheltered work and leisure activities during the first four months of the COVID-19 pandemic?
2. Were there fewer incidents and PRN medications in the first 5 months of pandemic compared to prior?

## Methods

A quantitative design was used, including two different data sources. The first data set includes the answers from a questionnaire (see Supplemental material for Appendix 1). The second data set includes reports from the deviation notice system at the Signo Vivo.

### Participants and setting

The setting is group home 1 at the Signo Vivo. Group home 1 was chosen because residents in this group home have a minimum of one-to-one staffing, and they have the most multiple and complex needs at the Signo Vivo. The group home 1 includes six houses, each for four or five residents; altogether twenty-eight residents. Twenty-six residents and their main workers were invited for participation. The age of the residents were between 24 and 88 years, with a mean of 47,63 years (SD = 14,31). All but one (of age 88) were between 24 and 65 years old. Two participants were not invited due to staff turn-over. Twenty-four residents accepted the invitation. All 24 residents communicate. However, communication capacities among the residents are widely heterogenous, including different use of sign language, braille, and verbal speech combined with sign language and/or braille. None of the participants were diagnosed with severe or profound ID. See Table 1 for resident information. Family members were not invited, because they had very limited contact with the residents during the pandemic lock-down.

**Table 1.** Participants, n = 24.

ID level	Mild 3	Moderate 12	Severe/profound 0	Unspecified 9
Communication	Read/write 4	Talk 7	Sparse verbal /don’t talk 13	
Autism	Yes 17	No 7	Don’t know 0	
ADHD	Yes 2	No 22	Don’t know 0	
Hearing	Deaf 11	Impaired 13		
Vision	Blind 1	Impaired 4		
Wheel chair	2	Muscle/skeletal 1		
Somatics	Circulair 4	Diabetes 1	Brain /Syndrome 16	Allergy/asthma 4
Mental disorder	Anxiety 4	Depression 2	Other 4	

The average time of the professional relationships between the residents and their primary worker was 6.7 years (SD 2.0 – 20.4 years). The average age of primary workers was 40.9 years. The primary workers had varied educational backgrounds, such as nurse, special need educator, preschool teacher, social pedagogue, occupational therapist, and nurse aid.

### Measures

A questionnaire was constructed, encompassing both check-off questions (number 1 – 4A), answered by the residents, and open-ended questions, which were answered by the main workers. The questions were constructed against the background of observations from workers observing less stress in residents. Stress was operationalised as *unease, sleep*, and *irritability* (Keane et al., 1994).

The deviation notice system at the Signo Vivo constitutes the second measure. Based on observations from workerss at the Signo Vivo, two domains of deviation notices were chosen: *staff safety* (violent behaviour towards staff members), and *PRN medication* (mostly sedatives). Data was extracted during three periods of time: 1. throughout 2019, 2. January 1 – March 31, 2020, and 3. April 1 – August 23, 2020.

### Procedure

The interviews were initially planned to be conducted by the first author face to face with the participants followed by a primary workers. However, due to infection control, this was not possible, as no visitors were allowed indoors. Hence, the primary worker for each resident co-operated as they wrote down the answers, as the participants (as mentioned above) communicate in largely different ways, and less than a handful are able to write.

Quantitative data were extracted from the deviation notice system at the Signo. This system gathers all notifications by house (house 1, house 2 and house 3). For the present study, the reports on *staff safety* and *PRN medication* were extracted from the deviation system for the three periods mentioned above, by the first author.

### Analysis

The quantitative data from the questionnaires, together with reports from the deviance report system at the Signo Vivo were analysed for frequencies (see Table 2). Regarding the comments from the workers and residents in the questionnaire, we wanted to find possible experiences of stressful situations for the residents, and also situations reported by workers as stress-reducing. Psychological stress is found to be a considerable problem for people with intellectual and developmental disability (Janssen et al., 2002; Bramston and Fogerty, 2000). Moreover, stress is found to be related to poor adaptive skills, and challenging behaviour in people with ID (Bramston and Fogerty, 2000; Bender et al., 1999). In order to systematise the comments, we used quantitative content analysis (Rourke and Anderson, 2004), as this is a suitable way to systematise the frequencies of certain phenomena. Stress may be observed as irritability, anxiety, unease, inadequate coping, and frustration (Allen, 2008; Keane et al., 1994). Hence, we looked for words in the comments from workers corresponding to terms mentioned above.

**Table 2.** The informants answers about unrest, sleep, irritability, work, and activities.

Group 1, Less stress or as usual, 19 residents	Unease	Less 7	As usual 11	More 1
	Sleep	Less 5	As usual 3	More 11
	Irritability	Less 6	As usual 13	More 0
	Miss leisure activities	Yes 5	No 9	Don’t know 5
	Miss sheltered work	Yes 1	No 7	Don’t know/do not work 11
Group 2, more stress, 5 residents	Unease	Less 0	As usual 0	More 5
	Sleep	Less 5	As usual 0	More 0
	Irritability	Less 0	As usual 0	More 5
	Miss leisure activities	Yes 5	No 0	Don’t know 0
	Miss sheltered work	Yes 5	No 0	Don’t know/do not work 0
	Back to work	As before 5	More 0	Less / want to quit work 0

All answers are from numbers of residents, 3 residents in group 2 did not answer this question.

### Ethical considerations

The study was approved by the Norwegian Centre for Research Data, NSD (ref. no. 513552). The Regional Ethical Committee for health research, REC, also approved the study (project number 197844). Adapted written information was provided for the participants, who gave their consent. For the participants in this study with dual sensory loss, more severe ID and mental and/or physical disorders, next of kin or the legal guardian consented. The participants in this study need highly facilitated communication. We therefore decided to ask the primary workers to co-operate with the participants in order to answer the questions, as the first author was hindered by infection control.

## Results

Nineteen residents reported that they were getting more rest or the same amount of rest compared to prior of the pandemic, and more or the same amount of sleep and less or the same amount of irritability. Five residents reported to be more stressed during the pandemic. They slept less, reported less rest and felt more irritable. Common for these residents was that they reported less rest linked to change of routines, even if the workers tried to find solutions similar to work and leisure activities. One of these five residents was very upset because his annual visit to a city he usually visits every year in May was cancelled. These five residents all felt bad about being out of both work and leisure activities.

The answers from questions 4 – 10 showed four categories of changes during the pandemic: *regular activities, social activities, personal routines*, and *interaction with the world outside*. Changes of regular activities included closedown or less work and activities, and that work and activities were brought home to the residents. Social activity changes included more time with staff members and fellow residents. Personal routines included more time for morning rituals, more time at home, more time spent on social media / internet and television, and less transportation. The last category – the outside world – included less contact with family, fewer excursions, more walking in the neighbourhood, and less shopping. Table 3

**Table 3.** Results from the quantitative content analysis.

Situations	Events	N Scores*
Stressful situations	Breach of routines, unpredictability	7
	Demands for task solving or activities	7
	Going to work	6
	Gatherings of people	5
	Getting ready for work	2
	Busy caregivers	1
	Shopping	1
	Being alone in own flat	2
	Infection control restrictions	3
Situations of down- regulation of stress	More time to relax	9
	Less demands for task solving or activities	7
	More time in the mornings	7
	More time with other residents	5
	More time with staff members	5
	More sleep	4
	Staying in well-known surroundings	1
	Less people around	1

* = number of comments from professional caregivers.

Data were extracted on the number of incidents of PRN medication and staff injuries which occurred for three different periods. The average number of reported incidents per week were calculated in order to compare the periods (see Table 4). Because of the small number of data points (Group home 1 has only six houses), analyses of possible significant differences between the three periods were not conducted.

**Table 4.** Frequencies of reports from the Signo Vivio deviation notice system.

Staff safety, incidents of staff injury						
House no.	Number of Weeks in 2019: 52	Average per week in 2019	13 weeks 2020: 1.1.-1.4	Average per week 1.1.-1.4 2020	21 weeks 2.4- 23.8 2020	Average per week 2.4.-23.8 2020
House 1	7	0.13	1	0.076	0	0
House 2	47	0.9	17	1.307	10	0.48
House 3	60	1.15	17	1.307	13	0.62
House 4	6	0.11	0	0	1	0.05
House 5	17	0.35	6	0.461	10	0.48
House 6	20	0.43	3	0.23	4	0.10
Total	157	3.07	44	3.38	38	1.81
Medication, if any (PRN medication)						
House no.	Number of Weeks in 2019: 52	Average per week in 2019	13 weeks 2020: 1.1.-1.4	Average per week 1.1.-1.4 2020	21 weeks 2.4- 23.8 2020	Average per week 2.4.-23.8 2020
House 1			0	0	0	0
House 2			15	1.15	1	0.05
House 3			14	1.08	15	0.71
House 4			0	0	11	0.52
House 5			0	0	0	0
House 6			0	0	0	0
Total	No data	No data	29	2.23	27	1.29

## Discussion

The main finding is that in the present sample, a majority of people with complex, multiple needs including ID, sensory loss, and impaired mental health, in one house at the Signo Vivo centre, experienced fewer stressful events, more rest and sleep, and more time with smaller groups of workers. More walking in the neighbourhood was reported as positive. Less contact with family and friends, less shopping and fewer excursions were reported as negative.

Compared to reports on the COVID-19 close-down from people with ID who live by themselves, or do not have multiple complex needs (presented in the introduction), the findings of this study suggest that giving services to people with ID who have multiple complex needs should include more person-centred care, taking more into account the needs for rest, for small groups of workers, and for user initiated activities.

It is an interesting finding that less activity has been positive for the majority of the participants. In Norway, inclusion in society with regard to both work and leisure activities has been an ideal since the deinstitutionalisation during the 1990s (Kielland, 2010; Tideman and Tøssebro, 2002). For most people with ID, living outside the institutions has led to a more meaningful life, including work and attending social and cultural arenas. User involvement for people with ID has been emphasised and developed after the large institutions for people with ID were closed down (Tideman and Tøssebro, 2002). However, a small minority of people with ID and multiple, complex needs (Rosengard et al., 2007) may experience cognitive overload, including persistent psychological stress, from living up to the ideal of a good life with many activities, work, and social events. A quote from one of the primary workers at House 1 at the Signo Vivo illustrates that the changes in spring 2020 were something that the workers had not expected:

*The user’s physical health improved a lot. There have also been many opportunities for building more robust relationships between this user and his workers. We used to think that work was so important to him, and that summer holidays were hard for him. But now that work has been closed for months, he has not talked about his job once, and is not interested when we ask. We have planned to reduce his work load after the close-down.*

This example illuminates a topic discussed in another study including users with multiple, complex needs (Hørsrud and Bakken, 2022). This study found that users in community residential services, who are provided with specialist habilitation services, profited from fewer activities and more time at home during the pandemic. Hørsrud and Bakken discuss how, for users with ID and complex, multiple needs, who usually follow daily schedules, scope for user involvement was limited (ibid.). For users with multiple, complex needs, including sensory loss, communication difficulties may negatively impact possible user-initiated activities. One of the respondents said: ‘*There has been more quietness and predictability, more time, fewer workers and fewer demands. He has been able to present his wishes more clearly.’*

The data from incidents including PRN medication and staff injuries supports the participants’ answers and primary workers’ comments in the questionnaires. Staff safety appeared to have improved during the lockdown. From January 1 until the lockdown in 2020, there were almost twice as many injury reports from staff as during the lockdown. However, in spite of the reports about less user stress for most of the participants in the present study, more of the staff members expressed doubts about altering daily schedules after the lockdown. Our clinical experience supports that these doubts exist, even when it is obvious that the user is stressed out. Keeping established work agreements may appear safer for the workers, even if the users communicate discontent about too many work hours, too much transportation, or too many leisure activities.

### Strengths and limitations

The strength of this study is a potential clinical relevance. The study revealed possible stressors that may be decreased by alterations in daily schedules and emphasis on smaller groups of staff members. Additionally, more time to rest, and more time in the mornings seemed to be reported as good for a majority of the participants.

The first and major limitation is that most of the participants co-operated with their workers when answering the questionnaires. We do not know how this situation may have impacted the answers. The second limitation the relatively low number of included users, and that they all live at the same place. The third limitation is that the small number of participants makes comparative analyses inadequate. However, there were distinct differences between pre-pandemic reports of incidents and those during the pandemic.

## Conclusions

Psychological stress is known to be negatively related to well-being in the general population. An easy and systematic tool was used for self-reporting elicited answers from all residents, despite multiple, complex needs. However, we do not know if answering the questions with a worker alongside has impacted the residents’ self-reporting.

The reports of caring for people with multiple, complex needs during the COVID-19 pandemic may be a basis for more research on stress in people with multiple, complex needs and how re-evaluation of daily schedules, organizing of rotation arrangements for staff members, and increased user involvement combined, can reduce stress.

## Supplemental Material

Supplemental Material - The effects of lockdown of work and activities for adults with multiple, complex needs including sensory impairments during the pandemic in 2020 Supplemental Material for The effects of lockdown of work and activities for adults with multiple, complex needs including sensory impairments during the pandemic in 2020 by Trine Lise Bakken in Journal of Intellectual Disabilities
