# Supplementary material for: The effects of lockdown of work and activities for adults with multiple, complex needs including sensory impairments during the pandemic in 2020
Source: J Intellect Disabil. 2024 Feb 2;28(2):578–87. doi: 10.1177/17446295241232030 (PMC11059831; doi:10.1177/17446295241232030)
Supplement: Supplemental Material - The effects of lockdown of work and activities for adults with multiple, complex needs including sensory impairments during the pandemic in 2020 [file sj-pdf-1-jld-10.1177_17446295241232030.pdf]

Appendix. Interview guide

1	Background information regarding participants and main professional caregiver
2	Communication capacities
3	When you have been at home, not at work or activities:<br>Have you felt more stress / unrest: Less / As usual / More / NA (no answer)<br>Sleep: Less / As usual / More / NA<br>Irritability: Less / As usual / More / NA
4 A<br><br><br><br>4B	Do you miss leisure activities? Yes / No / DK (don't know)<br>If Yes, what do you miss?<br>Do you miss going to work? Yes / No / DK (don't know)<br>If Yes, what do you miss?<br>What is good / bad about going to work?<br>Does something stress you out at work / leisure activities?
5	Do you want to work after the pandemic:<br>Like before / More / Less<br>Comments:
6	Please tell what you have done when not attending work / leisure activities:<br>Here are some alternatives (underline):<br>– TV / movies / TV Shows<br>– In bed<br>– Use of the internet<br>– Walking<br>– Being with staff members<br>– Being with other residents<br>– Other:
7	How have you experienced the mornings when not going to work?
8	Any other comments for main professional caregivers:
